# Air Stabilization of Li_7_P_3_S_11_ Solid-State Electrolytes through Laser-Based Processing

**DOI:** 10.3390/nano13152210

**Published:** 2023-07-29

**Authors:** Yannick Eatmon, Joseph W. Stiles, Shuichiro Hayashi, Marco Rupp, Craig Arnold

**Affiliations:** 1 Department of Chemical and Biological Engineering, Princeton Univeristy, Princeton, NJ 08544, USA; 2 Department of Chemistry, Princeton University, Princeton, NJ 08544, USA; 3 School of Integrated Design Engineering, Keio University, Yokohama 223-8522, Kanagawa, Japan; 4 Department of Mechanical and Aerospace Engineering, Princeton University, Princeton, NJ 08544, USA; 5 Princeton Materials Institute, Princeton University, Princeton, NJ 08544, USA

**Keywords:** laser-based processing, solid-state electrolyte, energy devices

## Abstract

All-solid-state batteries (ASSBs) that employ solid-state electrolytes (SSEs) have the potential to replace more conventional batteries that employ liquid electrolytes due to their inherent safety, compatibility with lithium metal and reputable ionic conductivity. Li_7_P_3_S_11_ is a promising SSE with reported ionic conductivities in the order of 10 mS/cm. However, its susceptibility to degradation through oxidation and hydrolysis limits its commercial viability. In this work, we demonstrate a laser-based processing method for SSEs to improve humidity stability. It was determined that laser power and scanning speed greatly affect surface morphology, as well as the resulting chemical composition of Li_7_P_3_S_11_ samples. Electrochemical impedance spectroscopy revealed that laser treatment can produce SSEs with higher ionic conductivities than pristine counterparts after air exposure. Further examination of chemical composition revealed an optimal laser processing condition that reduces the rate of P${}_{2}$S${}_{7}$${}^{4-}$ degradation. This work demonstrates the ability of laser-based processing to be used to improve the stability of SSEs.

## 1. Introduction

Despite their long-standing and leading position in the battery world, safety challenges associated with liquid- and gel-based lithium-ion batteries, such as flammability, dendrite growth, and thermal effects, have driven the transportation field to continue innovating to develop alternatives with increasingly more energy and power density while improving overall system safety [1,2,3,4]. Among the possible substitutes all-solid-state batteries (ASSB) employing solid-state electrolytes (SSE) have continued to garner attention from both the scientific and commercial sectors due to their compatibility with metallic lithium, increase in specific energy associated with lithium anodes, and inherent safety associated with its all-solid-state construction [1,5,6,7].

There are three main types of SSE: organic solid polymer electrolytes, hybrid/composite electrolytes and ceramic electrolytes. Each type has their advantages and disadvantages; however, ASSBs based on hard materials, such as ceramic electrolytes, are able to withstand large mechanical forces and high temperatures compared to their polymeric and composite counterparts and have been shown to better resist Li dendrite growth [8,9,10]. Furthermore, there are many types of ceramic electrolytes with their own advantages and disadvantages, allowing for the use of specific types based on the intended application.

These materials are perhaps even more promising from the electrochemical perspective as both oxide and sulfur-based ceramic electrolytes have demonstrated conductivities as high as, and in some cases, even greater than current liquid electrolytes; they enable cycle lifetimes of over 1000 cycles and allow the system to achieve higher operating temperatures for superior performance in high-power and fast-charging applications [11,12]. Furthermore, both oxides and sulfides exhibit negligible electronic conductivity, a wide electrochemical stability window, and chemical compatibility with high-energy cathode and anode materials.

Typically there is a trade-off between oxide- and sulfide-based ceramic electrolytes. Oxide SSEs exhibit increased resistance to oxidation and hydrolysis; for example, LLTO and LLZO are known for their stability when exposed to air and moisture [13]. Conversely, sulfide SSEs are prone to degradation by oxidation and hydrolysis due to stronger interactions between oxygen and other elements in the SSE. For example, in Li${}_{7}$P${}_{3}$S${}_{11}$, O${}^{2-}$ has stronger interactions with P${}^{5+}$ than S${}^{2-}$ does, which results in oxidation susceptibility. Sulfides have low grain boundary resistance (which aids in ionic conductivity) and mechanical softness [14,15,16]. Their soft nature allows for room-temperature densification, which aids in producing an intimate contact with electrode materials [17,18]. Additionally, sulfide SSE are believed to have higher ionic conductivity due to the larger size of the sulfide ion compared to that of oxygen, which increases the pathway available for ion migration [13].

In particular, Li${}_{2}$S-P${}_{2}$S${}_{5}$ mixtures have shown conductivities as high as 10${}^{-2}$ S/cm in bulk-processed glass–ceramic materials [1,19,20,21]. Despite the potential advantages of ASSBs, they still have not achieved widespread commercial implementation. There are a number of reasons for this, including their high cost of fabrication, poor mechanical properties, and extreme sensitivity to air and moisture [22,23,24]. There have been a myriad of different techniques reported in the literature to overcome these challenges [25]. Some of these include chemical substitutions or doping in SSEs, which result in improved stability [26,27,28,29,30,31,32,33,34,35]. Various solution-processing techniques have been explored to tackle issues such as production time and cost, achieving suitable Young’s moduli for good electrode–electrolyte contact and reducing electrolyte thickness in order to increase device energy density [1,19,36]. Laser-based SSE processing has also been explored in the past on different SSE types.

Pulsed laser deposition (PLD) has been used for preparing sodium thiophosphate, Li-V-S-O (LVSO), and garnet-type LLZTO films [23,37,38]. Recent work on LLZTO SSEs showed that laser sintering results in denser films compared to conventional furnace sintering [37]. PLD has also been used in the fabrication of 80Li${}_{2}$-20P${}_{2}$S${}_{5}$ SSE films [39]. This Li${}_{2}$S-P${}_{2}$S${}_{5}$ ratio typically results in the formation of Li${}_{3}$PS${}_{4}$. While nanoporous β-Li${}_{3}$PS${}_{4}$ has a respectable ionic conductivity, reported to be roughly 10${}^{-1}$ mS/cm${}^{2}$, it is surpassed by Li_7_P_3_S_11_, with reported ionic conductivities of 1–2 mS/cm${}^{2}$ [19,40]. This type of SSE uses a precursor ratio of 70Li${}_{2}$S-30P${}_{2}$S${}_{5}$ and produces both PS${}_{4}$${}^{3-}$ and P${}_{2}$S${}_{7}$${}^{4-}$ species. P${}_{2}$S${}_{7}$${}^{4-}$ has poor thermal stability and can thermally degrade to produce P${}_{2}$S${}_{6}$${}^{4-}$ [41,42,43]. This results in poisoning of the SSE, as the ionic conductivity of P${}_{2}$S${}_{6}$${}^{4-}$ is several orders of magnitude less than that of P${}_{2}$S${}_{7}$${}^{4-}$ [44]. It is crucial to control the amount of P${}_{2}$S${}_{6}$${}^{4-}$ present in SSE samples. In this work, we studied the effects of post-sintering CW-laser (continuous wave) processing on Li_7_P_3_S_11_ SSEs. We investigated the effect that post-sintering laser processing has on surface morphology, chemical composition, and ionic conductivity, as well as on oxidation and hydrolysis resistance.

## 2. Materials and Methods

The SSE used in this study was Li_7_P_3_S_11_:70Li${}_{2}$S-30P${}_{2}$S${}_{5}$. Li_7_P_3_S_11_ powder was purchased from MSE Supplies, stored under an argon atmosphere and used without any purification. The electrolyte powder was first packed into a 1/4″ pressing die and placed in a hydraulic press. A force of roughly 10.5 kN was applied to the powder for at least 20 min in order to densify the electrolyte powder into a rigid pellet. The use of short hold times occasionally resulted in the cracking of the pellet or powder flaking off of the pellet surface. The average density of the pellets was 1.93 g/cm${}^{3}$. Following pelletization, the samples were sealed in a container under argon and were placed on the laser stage (Figure S1). A thin aluminum disk was used as the substrate. A 1060 nm Nd;YAG CW laser was used to process the samples. The laser was programmed to pass over each sample in straight, parallel lines. The laser parameters used included the following: hatch spacing of 200 μm with laser powers from 10 W to 40 W and scanning speeds from 200 mm/s to 600 mm/s. The effective fluence used for laser processing thus ranged from about 16.5 J cm${}^{-2}$ to 200 J cm${}^{-2}$ (laser spot size of 100 μm). The effective laser power was adjusted using a filter. Characterization was then carried out using a 532 nm Horiba Raman Spectrometer, Thermo Fisher K-Alpha X-ray Photoelectron Spectrometer, and a Quanta 200 FEG Environmental-Scanning Electron Microscope (SEM). Stability measurements were conducted by placing a pellet in air at roughly 75% relative humidity for a predetermined amount of time (1–10 min). Electrolyte pellets were used to make symmetric coin cells with 0.1 mm indium sheets, which were used to reduce contact resistance between the pellet and electrode. The coin cells were then used in electrochemical impedance spectroscopy (EIS) measurements to determine the SSE’s ionic conductivity (Figure S1). EIS measurements were carried out using a BioLogic SP-150 Potentiostat.

## 3. Results and Discussion

**Figure 1:**
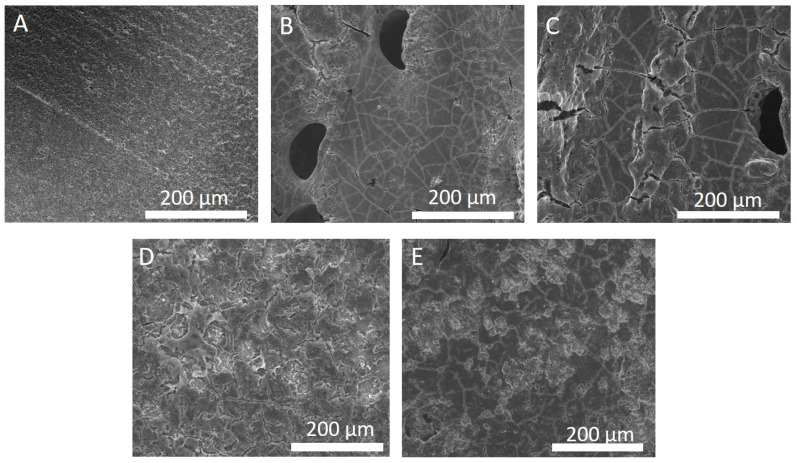
Scanning electron microscope images of electrolyte pellets after laser processing at a scanning speed of 200 mm/s with a hatch spacing of 200 μm across different laser powers (and fluences). (**A**) Pristine; (**B**) 10 W; (**C**) 20 W; (**D**) 30 W; (**E**) 40 W.

The energy supplied to samples during laser processing is directly proportional to laser power and is indirectly correlated with scanning speed. Therefore, by keeping the scanning speed constant and varying the laser power, we can observe the effect of varying energy on surface morphology. Figure 1 shows SEM images of pellets after laser treatment for different laser powers. Figure 1B,C show that a relatively low laser power of 10–40 W leads to cracking and the formation of holes/voids in the surface. Higher laser powers result in void-free surfaces (Figure 1D,E). Cracks are still present when higher laser powers are used, but to a lesser degree.

**Figure 2:**
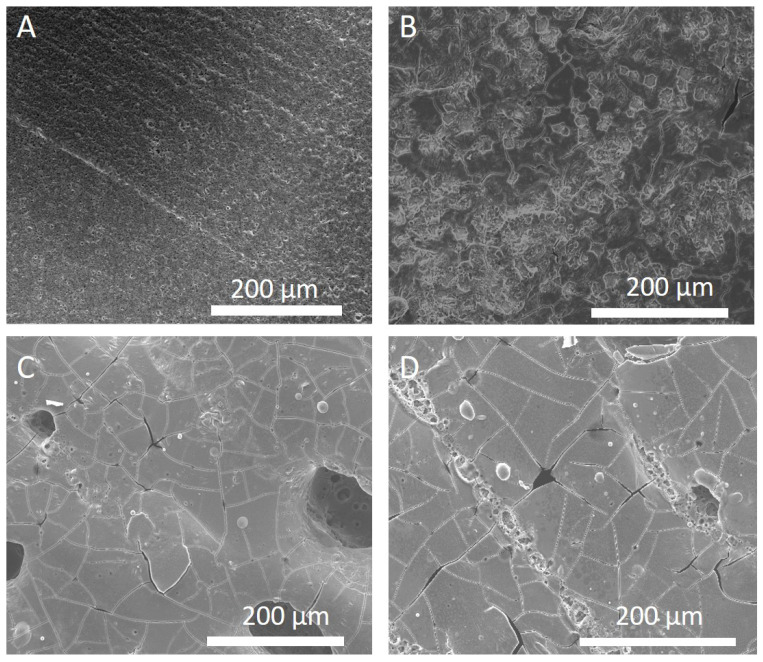
Scanning electron microscope images of electrolyte pellets after laser processing at a laser power of 40 W with a hatch spacing of 200 μm across different scanning speeds. (**A**) Pristine; (**B**) 200 mm/s; (**C**) 400 mm/s; (**D**) 600 mm/s.

In addition to varying laser power, we also investigated the role of scanning speed as a different variable to affect the energy supplied to a sample. Figure 2 shows the effect of scanning speed on the surface morphology of Li_7_P_3_S_11_ pellets. Figure 2C,D display SSE surfaces processed with relatively fast scanning speeds (400–600 mm/s). This resulted in the presence of cracks and voids. Conversely, Figure 2B shows that using a lower scanning speed of 200 mm/s produces a SSE surface with fewer cracks and no voids. This result indicates that using higher scanning speeds, which correlate with a decrease in the fluence, produces more cracks and voids in comparison to using lower scanning speeds and higher energy. Fluence is defined as the energy per unit area supplied to the surface of a material and is directly proportional to the ratio of laser power to scanning speed. Thus, based on the results from Figure 1 and Figure 2, we have determined that increasing the fluence, either by increasing power or lowering scanning speed, results in fewer cracks and voids on the surface of the SSE.

Laser irradiation with a CW laser causes heating of the sample, followed by a reduction in temperature due to air quenching. Relative to heating in a furnace, air quenching during laser treatment is a much faster process. Thus, the presence of cracks is attributed to faster quenching; large temperature gradients result in mechanical strain, which leads to cracking that propagates throughout the surface of the sample. It is believed that voids in the surface of the material are formed as a result of escaping argon gas from the bulk of the electrolyte. The solid-state electrolyte pellet is formed through the compression of fine powders, meaning that the pellet itself is somewhat porous with argon gas occupying the empty spaces between fine powder particles. Upon laser irradiation, the surface of the material rapidly heats up to form a molten laser melt pool. The high temperatures result in argon gas escaping from the bulk of the material through the laser melt pool. The combination of the rapid solidification of the melt pool with escaping argon results in the observed voids on the surface of laser-processed electrolyte samples.

**Figure 3:**
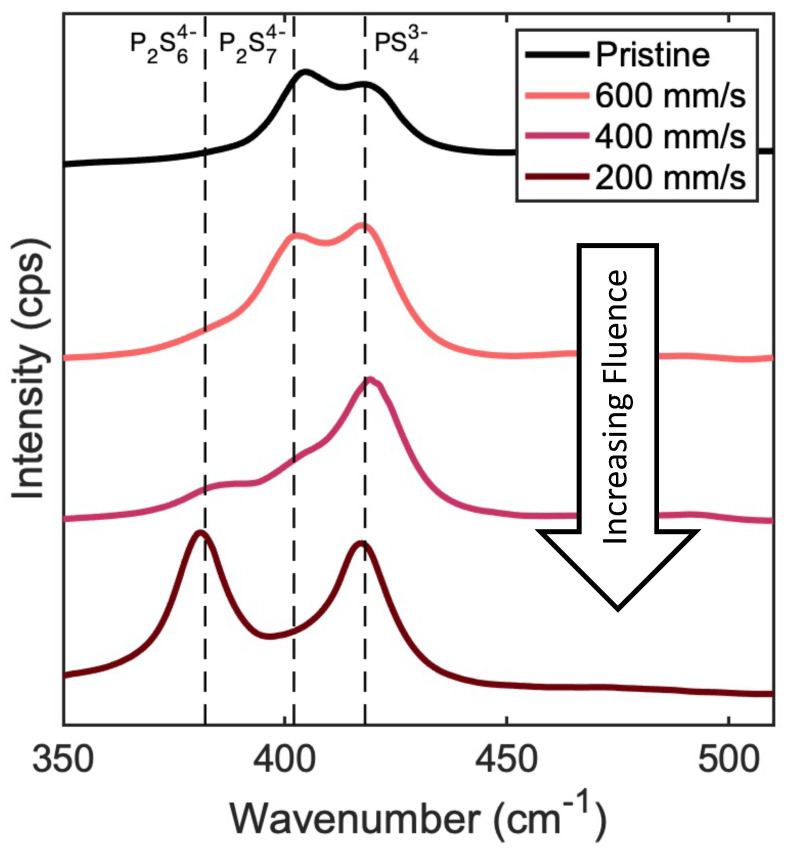
Raman spectra of Li_7_P_3_S_11_ pellets after laser processing at a laser power of 40 W with a hatch spacing of 200 μm with scanning speeds of 200 mm/s, 400 mm/s, and 600 mm/s. As scan speed decreases, the intensity of the P${}_{2}$S${}_{7}$${}^{4-}$ peak decreases, while that of P${}_{2}$S${}_{6}$${}^{4-}$ increases.

Based on SEM analysis, increasing laser processing fluence, by increasing laser power or lowering scanning speed, produces SSE surfaces with fewer voids. This suggests that higher fluences may be preferred. Reduced cracking and void formation will improve contact between the SEE and electrode, thus bolstering effective ionic conductivity and overall electrolyte performance. In addition to good surface morphology, maintaining the proper chemical composition is of obvious importance. To understand how laser treatment affects the chemical composition of these electrolytes, we conducted Raman spectroscopy on laser-processed SSE samples. Figure 3 shows Raman spectra for samples processed with a laser power of 40 W with varying scanning speeds. Li_7_P_3_S_11_ is characterized by the presence of two highly conductive phases, PS${}_{4}$${}^{3-}$ and P${}_{2}$S${}_{7}$${}^{4-}$ [45]. The peak around 420 cm${}^{-1}$ represents a vibrational mode for PS${}_{4}$${}^{3-}$ [45], which seems generally unaffected by laser treatment. This is, however, not the case for P${}_{2}$S${}_{7}$${}^{4-}$, with a vibrational mode that results in a peak around 406 cm${}^{-1}$. Laser treatment at a scanning speed of 400 mm/s and below results in significant P${}_{2}$S${}_{7}$${}^{4-}$ loss and P${}_{2}$S${}_{6}$${}^{4-}$ formation. P${}_{2}$S${}_{6}$${}^{4-}$, whose vibrational mode is represented by a peak at 385 cm${}^{-1}$, is a very poor ion-conducting material, with reported conductivities ranging from 10${}^{-7}$ to 10${}^{-10}$ S/cm${}^{2}$. The formation of P${}_{2}$S${}_{6}$${}^{4-}$ is mainly a result of thermal degradation of P${}_{2}$S${}_{7}$${}^{4-}$, which is more susceptible than PS${}_{4}$${}^{3-}$ to thermal effects [11]. Our results demonstrate that using faster scanning speeds for laser treatment will limit P${}_{2}$S${}_{6}$${}^{4-}$ formation and preserve P${}_{2}$S${}_{7}$${}^{4-}$ and PS${}_{4}$${}^{3-}$.

SEM and Raman analysis reveal a trade-off in SSE optimization performance. While higher fluence laser treatment results in more suitable surface morphology, i.e., fewer voids, it also results in thermal degradation. The trade-off between chemical composition and surface morphology is thus crucial to consider when determining appropriate laser processing parameters.

**Figure 4:**
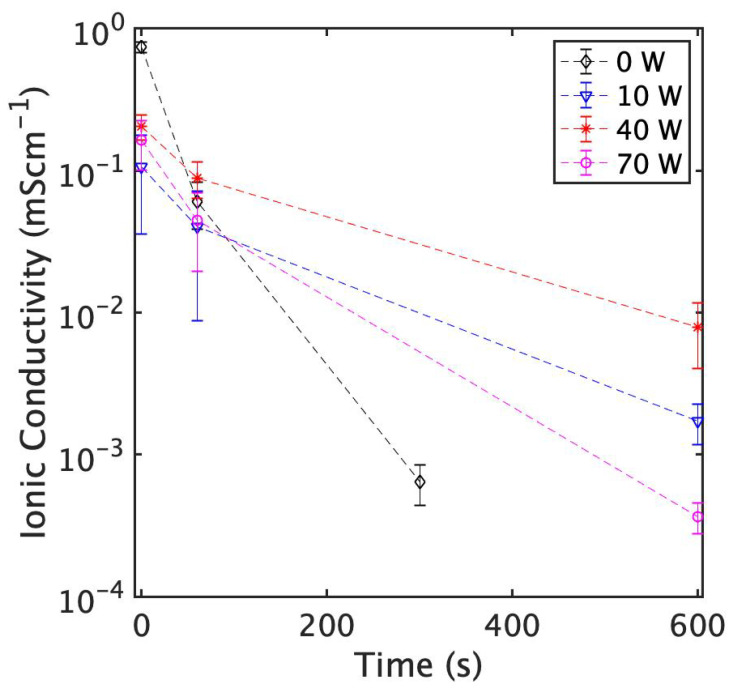
Ionic conductivity of Li_7_P_3_S_11_ pellets as a function of exposure time in air. Samples were treated using a scanning speed of 600 mm/s with varying laser powers.

**Figure 5:**
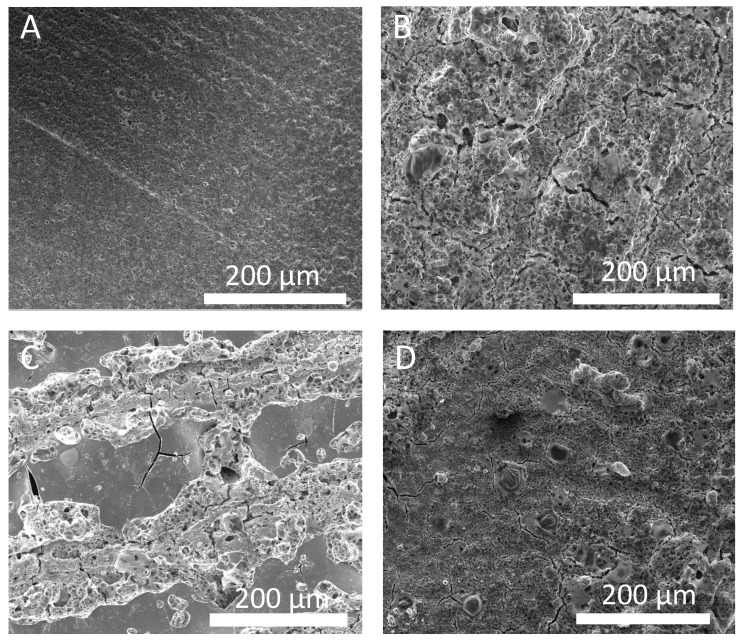
Scanning electron microscope images of electrolyte pellets after laser processing at a scanning speed of 600 mm/s with a hatch spacing of 200 μm across different laser powers. (**A**) Pristine; (**B**) 10 W; (**C**) 40 W; (**D**) 70 W.

Ionic conductivity is perhaps the most important metric regarding SSE; however, the stability of these electrolytes to degradation by oxidation and hydrolysis is also quite important to consider. Thus, we decided to not only study how laser treatment affects initial conductivity, but also determine what effects it has on the material’s ability to resist degradation through oxidation and hydrolysis. Guided by our Raman spectroscopy analysis, we decided to process samples for ionic conductivity measurements with a scanning speed of 600 mm/s and an unchanged hatch spacing of 200 μm. The laser power was scaled from 10 W to 70 W. Figure 4 shows the result of electrochemical impedance spectroscopy on laser-processed Li_7_P_3_S_11_ SSE pellets with varying levels of air exposure. SEM images of samples are shown in Figure 5. Across the laser-processed samples, those treated at 40 W displayed higher conductivities at each degradation time step. This is unsurprising given that these parameters resulted in the least amount of P${}_{2}$S${}_{7}$${}^{4-}$ degradation, as seen from our Raman analysis (Figure 3). This result suggests that an optimal laser processing parameter set exists which will maximize conductivity and stability. Relative to the pristine Li_7_P_3_S_11_ pellets, 40 W laser-processed samples display lower ionic conductivities, falling to approximately 0.7 mS/cm${}^{2}$. This initial reduction in ionic conductivity is attributed to the thermally driven conversion of some P${}_{2}$S${}_{7}$${}^{4-}$ to form P${}_{2}$S${}_{6}$${}^{4-}$, as well as cracking on the surface, which could affect contact with the electrode. Despite this initial drop in conductivity, after about a minute of air exposure, laser-processed samples begin to display comparable conductivities. At longer exposure times, 40 W laser-processed samples are more than an order of magnitude more conductive than pristine samples. Given that PS${}_{4}$${}^{3-}$ possesses relatively acceptable oxidation and hydrolysis resistance, loss of ionic conductivity due to air exposure is primarily a result of P${}_{2}$S${}_{7}$${}^{4-}$ degradation. Therefore, we postulate that under the right laser processing conditions, samples can become more resistant to P${}_{2}$S${}_{7}$${}^{4-}$ oxidation and hydrolysis, and thus more resistant to losses in ionic conductivity.

**Figure 6:**
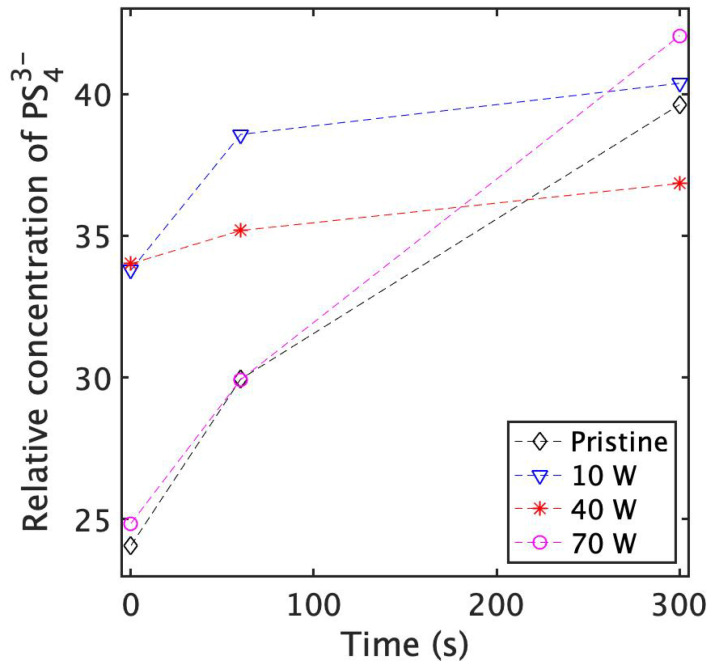
The intensity of P${}_{2}$S${}_{7}$${}^{4-}$, normalized to all sulfur species and derived from XPS analysis, evolves as a function of time under air exposure and laser processing power. Samples processed at 70 W seem to be similar to unprocessed samples; however, samples processed at 40 W experience less P${}_{2}$S${}_{7}$${}^{4-}$ loss. The seemingly high initial concentration of P${}_{2}$S${}_{7}$${}^{4-}$ is attributed to P${}_{2}$S${}_{6}$${}^{4-}$ production from high-power laser treatment and the overlapping of peaks for P${}_{2}$S${}_{7}$${}^{4-}$ and P${}_{2}$S${}_{6}$${}^{4-}$. Values shown in Table S1.

In support of our hypothesis, we used X-ray photoelectron spectroscopy (XPS) to observe how air exposure affects specific chemical groups. We chose to rigorously fit our XPS data using a two-species model for PS${}_{4}$${}^{3-}$ and P${}_{2}$S${}_{7}$${}^{4-}$/P${}_{2}$S${}_{6}$${}^{4-}$ [36,45,46,47,48,49]. The sulfur 2p signal for P${}_{2}$S${}_{6}$${}^{4-}$ is assumed to overlap greatly with that of P${}_{2}$S${}_{7}$${}^{4-}$. Figure S2 shows sulfur 2p XPS spectra for pristine Li_7_P_3_S_11_, where peak deconvolution allows us to integrate values for the S 2p^1/2^ and S 2p^3/2^ spin states for each species. Figures S3 and S4 show the effect of air exposure on S 2p and P 2p XPS spectra for pristine SSE samples and SSE samples laser processed at a high scanning speed across different laser powers. Across all power levels, the most noticeable change as a result of air exposure is a shift in the ratio of the two S 2p peaks. The change in the relative amounts of each phase due to air exposure is captured in Figure 6. By fitting our sulfur 2p XPS spectra, we can plot the area of the PS${}_{4}$${}^{3-}$ signal, normalized to the total area of all species. PS${}_{4}$${}^{3-}$ and P${}_{2}$S${}_{6}$${}^{4-}$ will not degrade in air in this timescale (Figure S5), which is in agreement with the literature. Thus, any increase in the relative concentration of PS${}_{4}$${}^{3-}$ is attributed to an actual decrease in P${}_{2}$S${}_{7}$${}^{4-}$ due to oxidation and hydrolysis.

This study revealed a non-linear relationship, where the “medium” laser power (40 W) resulted in the smallest change in the ratio of chemical compositions. This further suggests that there exists some optimal set of laser parameters that can minimize P${}_{2}$S${}_{7}^{4-}$ loss, hence improving stability towards oxidation and hydrolysis.

In order to better understand why some samples lose P${}_{2}$S${}_{7}$${}^{4-}$ at different rates, we returned to SEM analysis to try to explain the observed differences. Figure 5 shows the surface images of SSEs used for EIS and XPS studies. It has been shown that the laser sintering of SSEs can result in the densification of the material [37]. This leads us to believe that crack formation on the surface is also influenced by some densification of the material. Thus, we hypothesize that areas of the sample that had direct laser beam exposure act as blocking layers since they have a higher density than surrounding regions. This proposed blocking layer helps to reduce oxidation and hydrolysis in the bulk and near-surface by inhibiting H${}_{2}$O penetration. Electrolyte performance may also be influenced by surface roughness, which is unattainable from SEM images, but regardless, the results show that laser processing of electrolyte samples can lead to direct improvements in ionic conductivity after air exposure compared to untreated electrolyte samples under the same conditions. The optimal laser parameter set is therefore one that minimizes P${}_{2}$S${}_{6}$${}^{4-}$ formation while maximizing densification.

## 4. Conclusions

In this study, we investigated the effect of post-SSE-formation laser processing. We determined that laser fluence greatly affects SSE surface morphology and chemical composition. Through SEM analysis, we showed that decreasing fluence through a reduction in laser power or through an increase in scanning speed causes cracks and holes to form on the surface of SSE samples. Raman spectroscopy analysis, however, revealed that using slow scanning speeds (higher fluence) results in a reduction in the concentration of the highly conductive P${}_{2}$S${}_{7}$${}^{4-}$ phase. A trade-off, hence, exists between surface morphology and chemical composition from varying laser power and/or scanning speed. This trade-off was observed in the EIS aging study, where it was observed that using a “medium” laser fluence can maximize conductivity and stability relative to higher and lower fluences. Further analysis using XPS suggested that laser processing can reduce the amount of P${}_{2}$S${}_{7}$${}^{4-}$ lost through oxidation and hydrolysis. This can even be optimized through proper parameter selection. Finally, we returned to SEM analysis, where we hypothesized that densification resulting from laser treatment reduces the ability of H${}_{2}$O molecules to penetrate and hydrolyze the material. In this work, we demonstrated humidity stabilization through the post-formation laser processing of SSEs. We found that post-sintering processing can improve the humidity resistance of Li_7_P_3_S_11_ SSE and that there exists an optimal laser processing condition to do so. At slow scanning speeds or high laser power there is too much thermal damage, which results in deleterious effects to chemical composition and thus ionic conductivity. Conversely, at fast scanning speeds and low laser power there is widespread cracking, void formation, and insufficient densification, which also results in a reduction in ionic conductivity. Finding a balance is crucial for minimizing the drop in initial ionic conductivity and maximizing stability in air. Although these results are for SSE pellets, we envision the combination of this technique with other processing methods such as slurry casting.

## Data Availability

Not applicable.

## Supplementary Materials

The following supporting information can be downloaded at https://www.mdpi.com/article/10.3390/nano13152210/s1: Figure S1: (A) Image of portable chamber used for laser processing electrolyte pellets while under an argon atmosphere. (B) Image of a pellet after being laser processed in two perpendicular directions. (C) Image of a laser-processed pellet being used to make a symmetric (coin) cell; Figure S2: Deconvoluted X-ray photoelectron spectroscopy spectra of the sulfur 2p region of Li_7_P_3_S_11_ SSE samples. The figure shows the sulfur 2p ^1/2^ and ^3/2^ orbitals; Figure S3: Deconvoluted X-ray photoelectron spectroscopy spectra of the sulfur 2p region of Li_7_P_3_S_11_ SSE samples as a function of fluence used in laser treatment and exposure time in air; Figure S4: Deconvoluted X-ray photoelectron spectroscopy spectra of the phosphorous 2p region of Li_7_P_3_S_11_ SSE samples as a function of fluence used in laser treatment and exposure time in air; Figure S5: Raman spectra of Li_7_P_3_S_11_ pellets as a function of exposure time in air. Samples were processed at a laser power of 25 W with a scanning speed of 200 mm/s and a 200 μm hatch spacing. Table S1. Relative percentage of PS${}_{4}$${}^{3-}$ in laser-processed samples as a function of effective laser power and exposure time in air.
