# Supplementary material for: Air Stabilization of Li7P3S11 Solid-State Electrolytes through Laser-Based Processing
Source: Nanomaterials (Basel). 2023 Jul 29;13(15):2210. doi: 10.3390/nano13152210 (PMC10421269; doi:10.3390/nano13152210)
Supplement: Supplementary file 1 [file nanomaterials-13-02210-s001.zip › Laser Paper Supplementary Information Final.pdf]

Air Stabilization of $Li_7P_3S_{11}$ Solid State Electrolytes through Laser-Based Processing

Yannick Eatmon[a], Joseph W. Stiles[b], Shuichiro Hayashi[c], Marco Rupp[d], Craig Arnold[d,e]*

[a]Department of Chemical and Biological Engineering, Princeton University, Princeton, NJ 08544, USA
[b]Department of Chemistry, Princeton University, Princeton, NJ 08544, USA
[c]School of Integrated Design Engineering, Keio University, Yokohama, Kanagawa, Japan
[d]Department of Mechanical & Aerospace Engineering, Princeton University, Princeton, NJ 08544, USA
[e]Princeton Materials Institute, Princeton University, Princeton, NJ 08544, USA

Supplementary Information

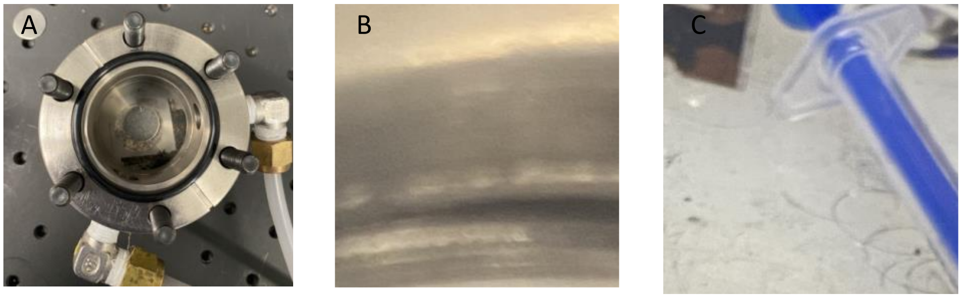

Figure S1. (A) Image of portable chamber used for laser processing electrolyte pellets while under Argon atmosphere. (B) Image of a pellet after being laser processed in two perpendicular directions. (C) Image of a laser processed pellet being used to make a symmetric (coin) cell.

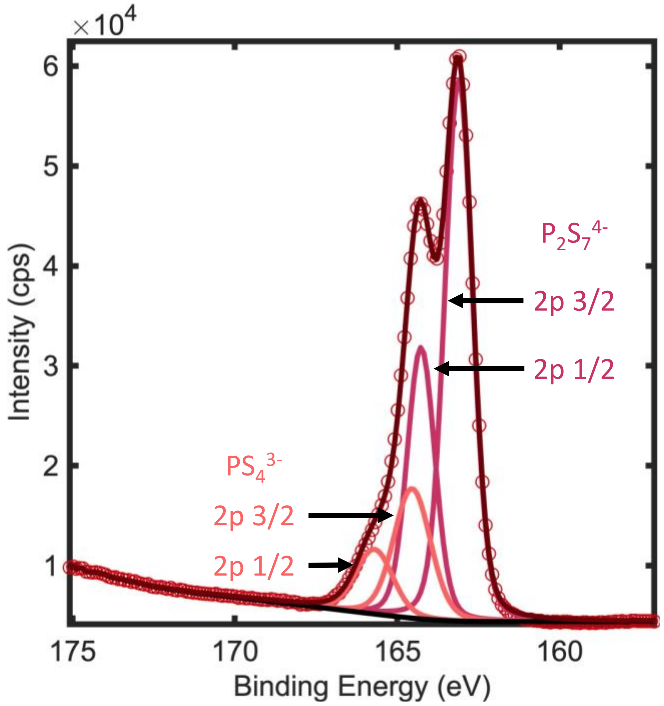

Figure S2. Deconvoluted X-ray Photoelectron Spectroscopy spectra of the Sulfur 2p region of $Li_7P_3S_{11}$ SSE samples. Figure shows the Sulfur 2p $^3/_2$ and $^1/_2$ orbitals.

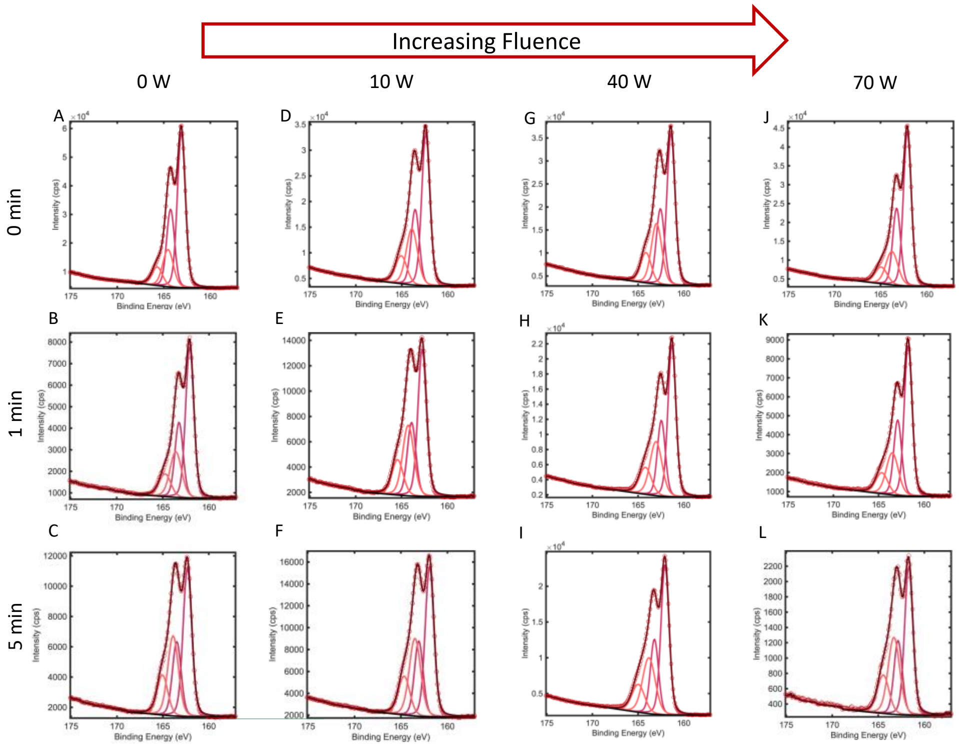

Figure S3. Deconvoluted X-ray Photoelectron Spectroscopy spectra of the Sulfur 2p region of $Li_7P_3S_{11}$ SSE samples. Spectra show the evolution of binding energies as a function of laser processing power and exposure time in air. (A-C) Unprocessed at (A) 0 min, (B) 1 min and (C) 5 min. (D-F) 10W at (D) 0 min, (E) 1 min and (F) 5 min. (G-I) 40W at (G) 0 min, (H) 1 min and (I) 5 min. (J-L) 70W at (J) 0 min, (K) 1 min and (L) 5 min.

	**0 W**	**10 W**	**40 W**	**70 W**
**0 min**	24.1%	33.8%	34.0%	24.8%
**1 min**	29.9%	38.6%	35.2%	29.9%
**5 min**	39.6%	40.4%	36.8%	42.1%

Table S1. Relative percentage of PS_4^{3-} in laser-processed samples as a function of effective laser power and exposure time in air. Relative percentages were calculated based on area under the curve calculations from deconvoluted S 2p XPS spectra (Figure S3). *Seemingly high initial concentration of $P_2S_7^{4-}$ is attributed to $P_2S_6^{4-}$ production from high power laser treatment and the overlapping peaks for $P_2S_7^{4-}$ and $P_2S_6^{4-}$.

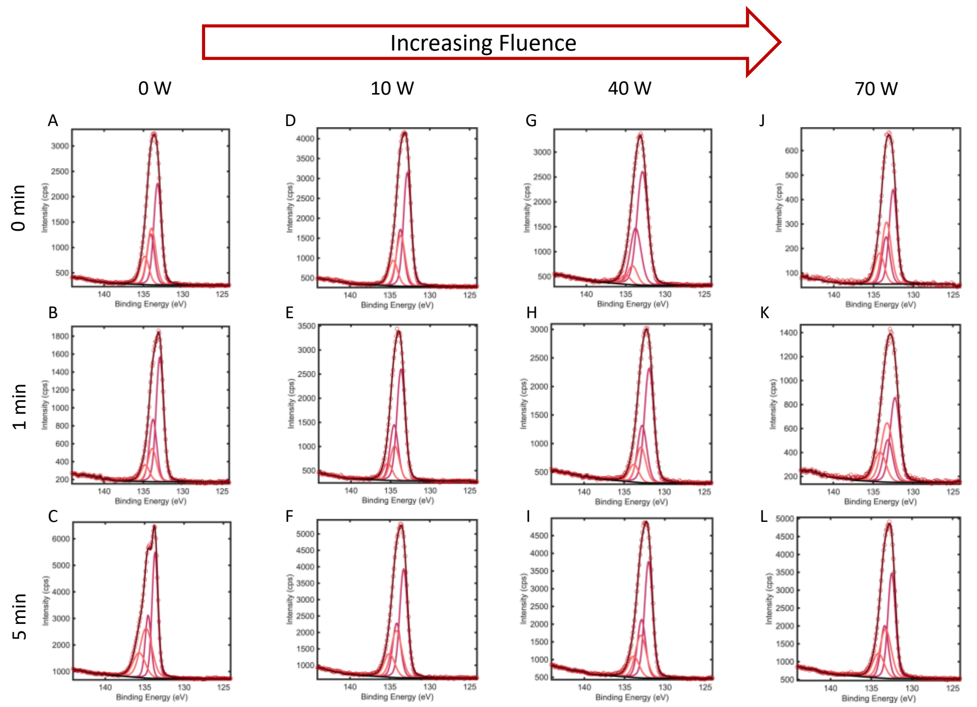

Figure S4. Deconvoluted X-ray Photoelectron Spectroscopy spectra of the Phosphorous 2p region of $Li_7P_3S_{11}$ SSE samples. Spectra show the evolution of binding energies as a function of laser processing power and exposure time in air. (A-C) Unprocessed at (A) 0 min, (B) 1 min and (C) 5 min. (D-F) 10W at (D) 0 min, (E) 1 min and (F) 5 min. (G-I) 40W at (G) 0 min, (H) 1 min and (I) 5 min. (J-L) 70W at (J) 0 min, (K) 1 min and (L) 5 min.

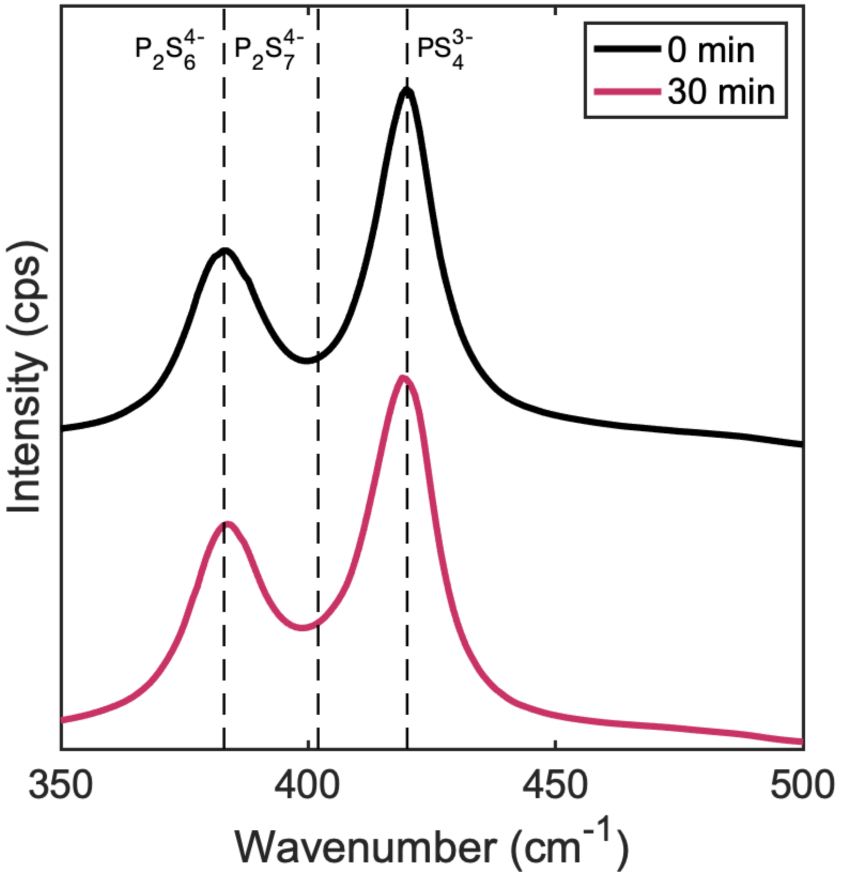

Figure S5. Raman Spectra of $Li_7P_3S_{11}$ pellets as a function of exposure time in air. Samples were processed at a laser power of 25W with a scanning speed of 200mm/s and a 200 μm hatch spacing**.**
